# EDCTP regional networks of excellence: initial merits for planned clinical trials in Africa

**DOI:** 10.1186/1471-2458-13-258

**Published:** 2013-03-22

**Authors:** George M Miiro, Odile Ouwe Missi Oukem-Boyer, Ousmane Sarr, Maerangis Rahmani, Francine Ntoumi, Keertan Dheda, Alexander Pym, Souleymane Mboup, Pontiano Kaleebu

**Affiliations:** 1 EACCR: The East African Consortium for Clinical Research, Uganda Virus Research Institute, Plot 51-59 Nakiwogo, Entebbe, Uganda; 2 CANTAM: The Central African Network for Tuberculosis HIV/AIDS and Malaria, Fondation Congolaise pour la Recherche Médicale, University Marien Ngouabi, Brazzaville, Republic of Congo; 3 WANETAM: The West Africa Network of Excellence for TB, AIDS and Malaria Laboratoire de Bacteriologie-virologie CHU Aristide Le Dantec, Universite Cheikh Anta Diop, Dakar, Senegal; 4 TESA: The Trials of Excellence for Southern Africa, Medical Research Council South Africa- TB Research Unit: Clinical and Biomedical, Durban, South Africa; 5 Centre de Recherche Médicale et Sanitaire (CERMES), Niamey, Niger; 6 The Institute for Tropical Medicine, University of Tubingen, Germany

**Keywords:** Regional networks, Health, Clinical trials, Research, Capacity-building, Africa

## Abstract

**Background:**

Achieving the Millennium Development Goals (MDGs) and combating hotspots with escalating but preventable communicable diseases remain major challenges in Africa. The European and Developing Countries Clinical Trials Partnership (EDCTP) intervened to combat poverty-related diseases including malaria, tuberculosis and HIV/AIDS, and to conduct multi-centre clinical trials and multi-disciplinary health research through an innovative model of regional Networks of Excellence (NoEs).

**Methods:**

We participated in a quasi-formative evaluation between October and December 2011 on the 4 regional-led research networks. These included the: Central Africa Network on Tuberculosis, HIV/AIDS and Malaria (CANTAM); East African Consortium for Clinical Research (EACCR); West African Network of Excellence for TB, AIDS and Malaria (WANETAM), and the Trials of Excellence for Southern Africa (TESA) launched between 2009 and 2010. We shared a participatory appraisal of field reports, progress reports and presentations from each network to jointly outline the initial experiences of the merits, outputs and lessons learnt.

**Results:**

The self-regulating democratic networks, with 64 institutions in 21 African countries, have trained over 1, 000 African scientists, upgraded 36 sites for clinical trials, leveraged additional € 24 million and generated 38 peer-reviewed publications through networking and partnerships.

**Conclusions:**

The shared initial merits and lessons learnt portray in part the strengthened capacity of these networks for improved research coordination and conduct of planned multi-center clinical trials in Africa. Increased funding by African agencies, governments and international health partners will ensure sustainability of these networks for research capacity development and demonstrate their commitment to achieving the MDGs in Africa.

## Background

The Millennium Development Goals (MDGs) are described as ambitious for sub-Saharan Africa and are a real barometer to assess countries' efforts towards improving the health of populations [1]. These universal goals target, among other priorities, poverty-related diseases such as tuberculosis, malaria and HIV/AIDS. Although Africa bears the greatest burden of these three major diseases with potential for global transmission, the continent is characterized by weak and under-resourced health infrastructure, health interventions inappropriate to the scale of the problem, and benefits of health not reaching those with the greatest disease burden [2,3]. In addition, African health research institutions are crippled by fragmentation, lack of coordination, diminishing critical mass of qualified African researchers, inadequate research infrastructure, and inconsistent and limited funding opportunities [4,5]. Such challenges hamper the contribution of African leadership to impact on research about diseases of global health importance.

**Figure 1:**
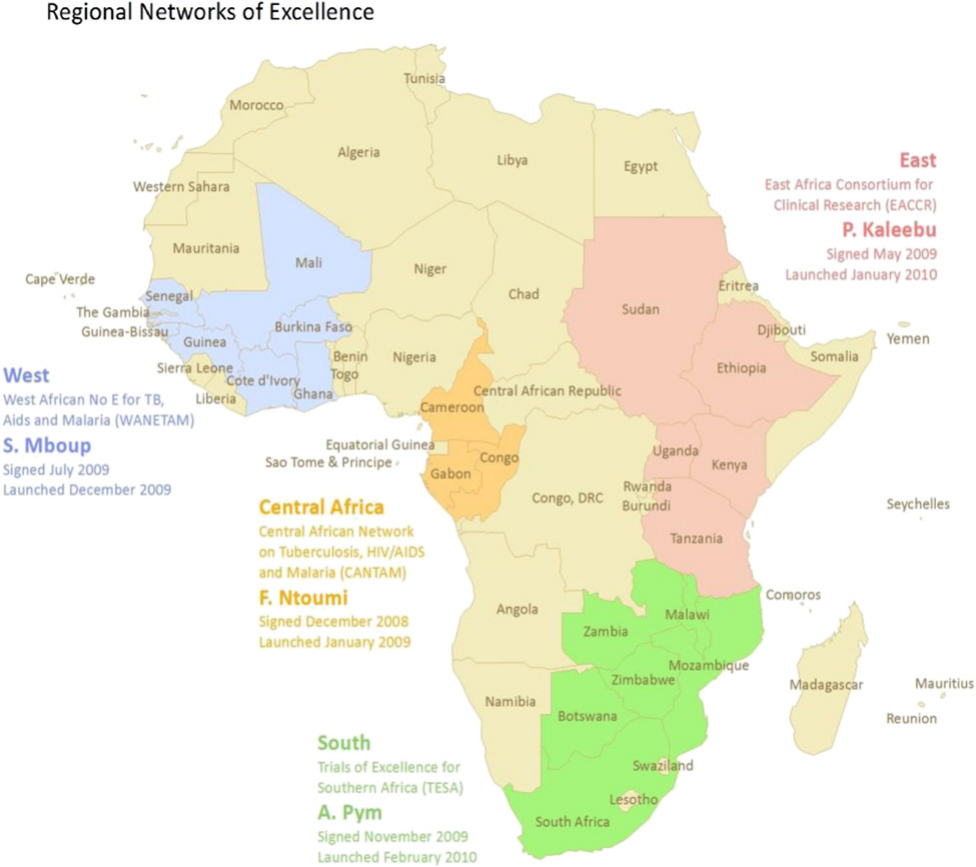
**Map of Africa showing the regional distribution and country composition of the four.** Regional Networks of Excellence. Legend: There are 64 institutions from 21 African countries within the 4 research networks

To address some of these challenges, the European and Developing Countries Clinical Trials Partnership (EDCTP), a European Union-funded and peer-review grant awarding agency [6], initiated the concept of the regional Networks of Excellence (NoEs) led by African professionals [7] to champion capacity development, research excellence and networking, in partnership with European member states while concurrently contributing to the MDGs. Some of the MDGs addressed by 2015 include MDG4: reducing child mortality; MDG6: combating HIV/AIDS, TB, malaria and other diseases, and MDG8: developing global partnerships. Expected benefits of the regional NoEs include: a) sustainable multi-site research coordination and grant management capabilities, b) generating capacity for up scaling the number of qualified African scientists and health practitioners, c) securing infrastructure, partnerships and funds capable of responding efficiently to regional diseases and threats through synergy and multi-disciplinary collaboration. The strategic investment and contribution by the EDCTP to the four NoEs (Figure 1) for the conduct of multi-centre clinical trials and health research in Africa deserves particular appraisal [8].

The history and features of these networks are unique [9]. How were the networks formulated? Which strategies were developed by each network to achieve their objectives? How have these networks interacted? And finally what are some of the lessons learnt through the NoEs’ programme? This joint introductory article addresses some of these key questions. Detailed results from each NoE will be subsequently published separately.

## Methods

We participated in a quasi-formative evaluation of the NoEs between October and December 2011 through participatory appraisal methods of direct observation, retrospective review of field reports, meeting presentations and formal reports to EDCTP at the 6^th^ EDCTP forum [10]. We outline the experienced effects and initial merits of the four African NoEs in terms of governance, baseline studies, capacity building, networking, research outputs, and ability to leverage further funding under this newly introduced model.

## Results

We outline below the results of each network (NOE) by the key reporting areas mentioned above.

### CANTAM (http://www.cantam.org)

#### A1. Project management

This West African-led network has 7 institutions from 3 African countries (Cameroon, Congo and Gabon, Table 1). Its governance relies on a steering committee, with one representative from each institution, responsible for all crucial decisions. This steering committee appoints a project coordinator at the secretariat in Congo who is in charge of the daily management of the network, assisted by two project managers from Gabon and Cameroon.

**Table 1:** Countries, institutions, diseases and members of the Networks of Excellence (NoEs) programme

**NoE (Project Coordinator) # of institutions**	**Country**	**Institution**	**Disease**			**Focal Person/PI**
			**HIV/AIDS**	**TB**	**Malaria**	
**CANTAM** (Francine Ntoumi) 7	Cameroon	Centre International de Référence Chantal Biya (CIRCB)	X			Odile Ouwe Missi Oukem-Boyer
		University of Yaoundé I		X	X	Rose Leke, Veronique Penlap
		University of Buea	X		X	Eric Achidi, Peter Ndumbe
		OCEAC			X	Parfait Awono
	Congo	University Marien Ngouabi	X	X	X	Francine Ntoumi, Obengui
		CERVE	X		X	Mathieu Ndounga
	Gabon	Medical Research Unit of the Albert Schweitzer Hospital	X	X	X	Saadou Issoufou
**EACCR** (Pontiano Kaleebu) 34 (represented here by 17 leading institutions	Sudan	Institute of Endemic Diseases (IEND), University of Khartoum		X	X	Mukhtar Maowia
	Ethiopia	Armauer Hansen Research Institute (AHRI)	X	X		Abraham Aseffa
	Uganda	Uganda Virus Research Institute (UVRI)	X			Edward Katongole-Mbidde, Jonathan Kayondo, Emily Nyanzi
		Medical Research Council (MRC) Unit on AIDS in Uganda	X			Alison Elliott, Heiner Grosskurth
		Makerere University and Infectious Disease Institute	X		X	Elly Katabira, Andrew Kambugu
	Kenya	Kenya Medical Research Institute (KEMRI)-Wellcome Trust Research Program			X	Norbert Peshu, Peninah Soipei Menza
		Kenya Medical Research Institute (KEMRI)-Walter-Reed Project	X		X	Bernhards Ogutu
		Kenya Medical Research Institute (KEMRI)-Centre for Global Health Research in partnership with CDC	X	X	X	Kayla Laserson
		Kenya AIDS Vaccine Initiative (KAVI)/University of Nairobi	X	X		Walter Jaoko, Omu Anzala
		Maseno University			X	Ayub Ofulla
	Tanzania	Kilimanjaro Christian Medical Centre (KCMC)/Kilimanjaro Clinical Research Institute (KCRI)	X	X	X	Gibson Kibiki, Frank Mosha, Reginald Kavishe
		Ifakara Health Institute (IHI)	X	X	X	Salim Abdulla, Seif Shekaleghe, Salim Nahya
		National Institute for Medical Research (NIMR)-Mwanza	X			John Changalucha, Mark Urassa
		National Institute for Medical Research (NIMR)-Tanga Research Centre			X	Martha Lemnge
		National Institute for Medical Research (NIMR)-Mbeya Medical Research Programme	X	X		Leonard Maboko, Lucas Maganga
		Muhimbili University of Health and Allied Sciences	X	X	X	Eligius Rwamuya, Joyce Masalu
		National Institute for Medical Research (NIMR) -Muhimbili	X	X		Mwele Malecera, Sayoki Mfinanga, Bernard Ngowi
**TESA** (Alexander Pym) 10	Botswana	Botswana Harvard HIV/AIDS Partnership (BHP)	X	X		Rosemary Musonda
	South Africa	South African Medical Research Council: Tuberculosis Research Unit (TBRU)	X	X		Alexander Pym
		Stellenbosch University Immunology Research Group (SUN-IRG)	X	X		Gerhard Walzl
		University of Cape-Town Lung Institute (UCT-LUNG)		X		Keertan Dheda
		University of Cape Town- Division of Clinical Pharmacology (UCT-PHARM)	X	X		Helen McIlleron
	Zimbabwe	University of Zimbabwe College of Health Sciences (UZ-CHS)	X			Lynn Zijenah
		Biomedical Research and Training Institute (BRTI)	X	X		Peter Mason
	Zambia	University of Zambia –University Teaching Hospital (UN-ZAM)	X	X	X	Peter Mwaba, Duncan Chanda
	Malawi	Malawi College of Medicine (Malawi- CoM)	X		X	Newton Kumwenda
	Mozambique	Centro de Investigacao em Saude da Manhica (CISM)	X	X	X	Eucebio Macete
**WANETAM** (Souleymane Mboup) 13	Senegal	Laboratory of Bacteriology-virology, A. Le Dantec Hospital (UCAD)	X	X		Souleymane Mboup
		Institut Pasteur, Dakar			X	Aissatou Toure
	The Gambia	National Public Health Laboratories	X			Makei Taal
		Medical Research Council, Fajara The Gambia	X	X	X	Tumani Corrah/Martin Antonio
	Guinea Bissau	Project de Saude de Bandim		X		Paulo Rabna
	Nigeria	College of Medicine University of Ibadan		X		Aderemi Kehinde
		Nigerian Institute of Medical Research		X		Oni Idigbe
		Innovative Biotech	X			Simon Agwale
	Ghana	Korle-bu Teaching Hospital University of Ghana Medical School		X		Audrey Forson
		Noguchi Memorial Institute for Medical Research			X	Kwado Koram/Nancy Duah
	Mali	Malaria Research and Training Center, Faculty of Medicine Bamako			X	Ogobara Doumbo/Mahamadou Thera
	Burkina Faso	Centre National de Recherche et de Formation sur le Paludisme, Ouagadougou			X	Sodiomon Sirima/ Issa Nebie
		Centre Muraz	X	X		Jean B Ouedraogo/ Nicolas Meda

#### A2. Baseline studies

The sister institutions in Congo and Cameroon have developed assessment surveys, while Gabon plays a leading role, with the Medical Research Unit in Lambaréné, already conducting clinical trials on malaria. By combining initial baseline studies and senior fellowship projects awarded to NoEs, CANTAM is better prepared to conduct future clinical trials in Cameroon (around Buea for HIV/AIDS, in Yaounde and Mbalmayo for tuberculosis, and in Niete and Mutengene for malaria) and Congo (all sites are in Brazzaville).

#### A3. Capacity building

**Figure 2:**
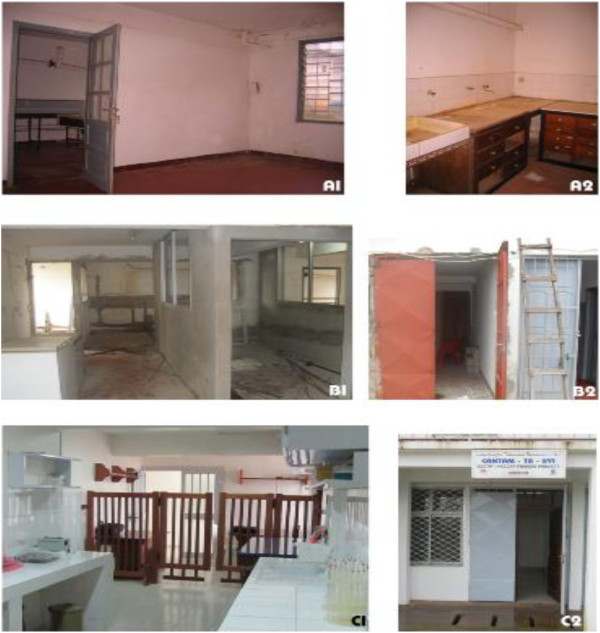
**An example of laboratory refurbishment in Central Africa (University of Yaoundé 1, Cameroon).** Legend: Old laboratory of the University of Yaoundé I, given to CANTAM in 2009 (A1, A2), laboratory under renovation in 2009-2010 (B1, B2); and refurbished laboratory with EDCTP/NACCAP funds (C1, C2) in 2011

Concerted effort has been put into capacity building: laboratory upgrades (at the University Marien Ngouabi and Makelekele hospital in Brazzaville, Congo and Buea and Yaoundé I universities in Cameroon; Figure 2); long –term training of masters, PhD, post-doctoral and medical students and cross-cutting specific and short-term courses, including grant writing, ethics and Good Clinical Laboratory Practice (GCLP) (Table 2). Long-term training and appropriate mentorships are essential, especially in Congo, where there is an acute shortage of human resources in all disciplines [11].

**Table 2:** Selected outputs from the Networks of Excellence (NoEs) by the end of 2011

**NoE**	**Type of training (number of participants are listed)**						**Funds (€) leveraged**	**Number of publications #**	**Number of sites upgraded**
	Short term*	**Diploma/BSc**	**MSc**	**PhD**	**Post Doc**	Fellowship**	-	-	-
CANTAM	83	-	6	8	-	4	1,152,360	8	4
EACCR	169	-	24	-	-	4	1,400,000	1	19
TESA	539	3	12	5	3	2	20,000,000	29	10
WANETAM	175	-	2	1	-	2	1,706,000	0	3
**Total**	**966**	**3**	**44**	**14**	**3**	**12**	**24,258,360**	**38**	**36**

* these include: GCP, GCLP, laboratory techniques, IT, ethics, grant writing, epidemiology, biostatistics, molecular biology, immunology, public health, clinical trials, parasitology and entomology.

** these are EDCTP sponsored senior fellowships which are affiliated to the NoEs.

# Selected publications have been referenced below due to space considerations but the full list of all publications is available on request.

#### A4. Networking

CANTAM has developed relationships within its network, and with local, regional and international organizations, which have resulted into field mentorships, shared training and joint seminars. Although North–south partnerships are essential in education (via ‘sandwich’ PhD programmes), technology transfer and joint grant writing, special attention has also been given to South-South partnerships, with emphasis on interactions with other networks for complementarity, synergy and greater impact. For example, EACCR facilitated short courses and mentorships for about 10 CANTAM students.

#### A5. Advocacy

EDCTP and CANTAM have been promoted through publications, international conferences and meetings, and networking with foreign and other organizations. Publications from CANTAM are increasing its visibility [11,12]. In Congo, Cameroon, and very recently in Gabon, EDCTP site visits were excellent opportunities for high-level advocacy and resource mobilization among prime ministers, ministers of higher education and of public health from each country.

#### A6. Funding

CANTAM received initial funding of € 2,997,644 from the EDCTP and Netherlands-African Partnership for Capacity development and Clinical interventions Against Poverty-related diseases (NACCAP). Through the EDCTP, additional funding has been awarded to CANTAM-affiliated institutions, including three senior fellowships linked to NoEs, a re-entry grant, and two grants for bioethics and regulatory strengthening. In addition, Total Congo agreed to support research on malaria and severe diarrhea. Paraxel plans to conduct a clinical trial in one of CANTAM's malaria sites in Cameroon. Finally, an EDCTP-sponsored clinical trial is being conducted in Gabon as an integrated project on malaria. Overall, CANTAM received € 5,238,000, including additional funds leveraged, for preparing clinical trial sites and conducting clinical research/trials, of which 78% is from EDCTP (Table 2).

### EACCR (http://www.eaccr.org)

#### B1. Project management

This East African-led network has 34 regional institutions from Kenya, Tanzania, Uganda, Sudan and Ethiopia (Figure 1, Table 1) and 7 northern partners from Europe. Governance is structured regionally into 4 coordinating centers (nodes): malaria (Kenya), TB (Tanzania), HIV (Uganda) and training (Tanzania), which report to the overall project coordinator. The coordinator reports to an independent steering committee (with directors of leading regional institutions and representatives of northern partners) and to EDCTP. Meetings occur regularly through teleconferences, and face-to-face interaction.

#### B2. Baseline studies

Nodes conducted site assessment surveys on existing regional capabilities for training/mentoring and also prioritized infrastructural upgrades among 17 sister institutions for research and health.

#### B3. Capacity building

#### Short-term training

Four accessible user-friendly electronic learning research modules were developed and uploaded onto the EACCR website in partnership with the University of Oxford, Global Health Trials, World-Wide Anti-malarial Resistance Network (WWARN), and the Bill and Melinda Gates Foundation. Plans are underway to translate the modules into French for improved accessibility and greater impact. EACCR also conducted short courses in epidemiology, medical statistics, clinical trial monitoring and tropical immunology in partnership with the London School of Hygiene and Tropical Medicine (LSHTM), University of Cambridge and the Wellcome Trust (Table 2). Thus, regular courses are available at a highly subsidized fee or through partial competitive scholarships.

#### Long-term training

Four competitive EDCTP senior-fellowships were secured for promising research in Malaria (1), TB (1) and HIV/AIDS (2) between November 2009 and November 2010. In addition, 24 masters research fellowships have been sponsored (Table 2), 7 of which are pursued online.

#### Reciprocal clinical monitoring scheme

More than 20 regional monitors are conducting cross-site paired mentoring visits using standardized operating procedures. Shared best clinical monitoring practices are observed by new/upcoming monitors paired to experienced monitors [13]. Consultancy services are available at a negotiable fee for tailored trial training and monitoring.

### B4. Networking

Participatory evaluation of the quality and bioethics of 2 research-monitoring schemes in East Africa and Asia is underway in partnership with WWARN and LSHTM. Furthermore, three EACCR’s fellows on masters degree training secured more research funding through highly competitive scholarships from a Wellcome Trust Consortium-Training Health Researchers in Vocational Excellence THRiVE (http://www.thrive.or.ug).

### B5. Advocacy

EACCR members shared thirty scientific presentations and abstracts at international meetings. This group interacted with other consortia such as Tuberculosis Vaccine Trials in Europe and Africa, TB-TEA (http://www.mpiib-berlin.mpg.de), CANTAM and THRiVE. In addition, contacts were initiated with policy-makers of the East African Community by EDCTP [14].

### B6. Funding

EACCR is funded by EDCTP with co-funding from NACCAP and the Medical Research Council-United Kingdom (MRC-UK) at a level of € 3.5 million. About € 1.4 million in additional funds have been leveraged from other sources, such as Global Health Trials, WWARN, Wellcome Trust and the International Association of National Public Health Institutes (Table 2). To sustain its activities, this network has submitted for funding at least 5 collaborative grants on research coordination, HIV prevention research, and rapid TB diagnostics.

## TESA (http://www.tesafrica.org)

### C1. Project management

This South African–led network has 10 institutions across 6 countries including Botswana, Malawi, Mozambique, South Africa, Zambia and Zimbabwe, supported by the EDCTP through the South African Medical Research Council (SA-MRC) TB Research Unit. TESA currently supports, on a part- or full- time basis, between 40–50 scientists, clinicians, nurses and laboratory technologists.

### C2. Baseline studies

Through the network’s capacity development strategy, TESA-network institutions have initiated several HIV, TB and malaria studies (some of which have been published [15-22]), either at individual sites or jointly within multiple sites. These studies include:

•Changes in plasma cytokine levels over time during chronic asymptomatic HIV-1c Infection, an indicator of disease progression

•Rifaquin trial in newly diagnosed TB cases, Pharmaco-Kinetic studies, and molecular studies using isolates from cases of treatment failure or re-infection, and suspected multi-drug resistant-TB [16,18,20]

•HIV-1 incidence rate among patients with sexually transmitted infections

•Diagnostic and biomarker studies in those patients with suspected TB

•Epidemiological, microbiological, clinical and socio-economic studies of re-treated TB patients

•Intensive case finding to determine the prevalence of drug sensitive and resistant-TB among those patients with suspected TB

•Accuracy and impact studies of Gene Xpert and newer diagnostic techniques including the urinary lipoarabinomannan strip test [15,17,19,21]

•Studies about the burgeoning drug-resistant TB epidemic and therapeutically destitute cases of drug resistant TB

### C3. Capacity building

Following advertisement of courses on the TESA website, trainees and students were selected based on the institutions’ internal training policies, research projects and availability of research grants. Within the past two years more than 500 clinical research staff affiliated to TESA received short-courses training in internal auditing and quality control, International Air Transport Association-IATA training, HIV, TB and malaria laboratory diagnostic techniques, and methodological courses including the epidemiological, clinical and operational research course; qualitative research methods course; information technology, software training; workshops on Good Clinical Practices and bioethics; and seminars on scientific and grant writing skills, and biostatistics (Table 2).

As part of TESA’s long-term capacity development program, 25 scientists are studying in various universities at masters, PhD and post-doctoral level (Table 2).

Three TB, HIV and malaria laboratories within TESA institutions have been accredited and a further three laboratories are in the process of accreditation. Two research training facilities and one clinical research site have been upgraded and most laboratories were refurbished with basic equipment and information technology support systems.

### C4. Networking

The networking within TESA has taken various forms including, i) student visits and attendance in cross country courses, ii) scientific presentations at national and international conferences and forums iii) attendance of regular national and regional technical meetings by investigators with the national departments of health, science councils, and academia, iv) linkages with national regulatory authorities and v) liaisons with European science councils, pharmaceuticals, donors and funding agencies.

### C5. Advocacy

The design and operationalization of the TESA Website in May 2010 facilitated above networking activities, communication and circulation of information about activities and courses, and linkage with international organizations.

### C6. Funding

TESA received € 2.3 million in November 2009 from EDCTP, followed by additional in-kind contribution of € 280,000 from SA-MRC. However, most TESA sites have since developed new research proposals, which to date have attracted an additional ~ € 20 million in research grants and contracts (Table 2).

## WANETAM (http://www.edctp.org/project_profiles.245.0.html)

### D1. Project management

WANETAM established 6 Work Packages (project management, training, networking, HIV-related training and survey, TB-related training and survey, and malaria related training and survey). Work package leaders and the project manager constituted the steering committee, chaired by the project coordinator. This committee meets every other month by teleconference to review progress in capacity-building and training. The coordinator is responsible for submitting annual technical and expenditure reports to the EDCTP. The responsibility for individual projects (resources, research and meeting milestones) was devolved to site Principal Investigators, who interact directly with Work Package leaders to produce written reports. WANETAM also has an independent advisory board, whose role is to advise the steering committee on the evolution of important activities.

### D2. Baseline studies

WANETAM did not initially include baseline studies. However, through the second annual meeting, submission of a revised work plan for year 3 and the subsequent re-allocation of funds, the principal investigators agreed to carry out HIV/AIDS, tuberculosis, and malaria baseline surveys as a sustainability strategy.

### D3. Capacity building

From January 2011 to June 2011 WANETAM trained 175 West African junior and senior scientists in clinical trial-related topics (Table 2) and disease-specific laboratory training organized by different partner institutions. Moreover, laboratories at Korle-bu (Ghana), and Guinea-Bissau have been refurbished. Specific equipment has been purchased for TB-associated laboratories while other laboratories have received equipment for hematology or biochemistry studies.

### D4. Networking

The secretariat has hosted two annual meetings of the principal investigators, which addressed the critical challenges that WANETAM has encountered (such as the initial omission of baseline studies and sustainability). The group has also established exchange and mentorship programmes between institutions. Additional networking has led to more funding through WANETAM Plus (constituted by WANETAM-TB institutions plus other institutions from Benin, Mali and Burkina Faso), and WAPHIR (West African Platform for HIV Intervention). Furthermore, joint study proposals have been submitted to the Trilateral Initiative (Germany, France, and WANETAM), and Framework Programme 7 of the European Commission grant call.

### D5. Advocacy

WANETAM created an efficient and reliable communication environment. Through a web-based platform called Basecamp, members can share information of interest, work on the same document, and discuss activities of the consortium (https://wanetamproject.basecamphq.com). Recently, the consortium has implemented a Voice on IP teleconference system allowing free communication among the partners. Prior to this, WANETAM created its website which is open to the public. In addition to the above communication strategies, WANETAM distributes regular newsletters.

### D6. Funding

Overall, the EDCTP contributed € 3,499,921. Of this, NACCAP, MRC-UK, and MRC-Gambia contributed € 1,000,000, € 406,000, and € 300,000 respectively.

## Discussion

The 4 NoEs described above, with 64 institutions from 21 African countries are African-led, multi-disciplinary, and multi-disease-oriented (Figure 1, Table 1). They are comparable to the subsequent Wellcome Trust initiative comprising 7 consortia which interlink 52 institutions from 18 African countries [23,24]. In contrast, the Malaria Capacity Development Consortium, Multilateral Initiative on Malaria and the ALPHA network have a single-disease orientation [25-27].

Robust and democratic governance structures are essential for the success of such networks [24]. The EDCTP NoEs have created described governance structures for self-regulation. The initial achievements and lessons learnt are discussed below. It is challenging to devise generic indicators for monitoring and evaluating capacity building since each project is unique [28]. Like other consortia [25-27], we predominantly referred to initial output-based (lower process) indicators from a donor reporting format such as number of fellowships sponsored, number of participants trained (Table 2), and number of facilities upgraded. We also attempted to include higher outcome indicators [5,25,27,28] such as amount of additional funds leveraged from alternative sources and number of publications. Over time, we shall track and include the number of PhD and doctoral fellows retained in Africa, and the number of policy outcomes. This paper contributes to further discussion of suitable indicators of progress for inclusion in subsequent monitoring and evaluation initiatives.

### Project management

Each network has a governance structure for better research coordination and resource sharing across Anglophone, Lusophone and Francophone countries in Africa. Another distinct feature of these NoEs is that consultative strategic planning processes were self-led rather than being dictated by the EDCTP. Thus, each NoE and partner institutions had the flexibility to adopt appropriate research capacity development activities well aligned to their preferences.

### Baseline studies

The NoEs opted to conduct baseline studies for two main reasons linked to sustainability strategies. First, they constitute a practical prerequisite for the conduct of clinical trials in future. Second, in terms of visibility, these studies are publishable aspects of the research activities for each NoE.

### Capacity building

NoEs have invested substantially in clinical trial infrastructure and in education, through specific or crosscutting short-term training, long-term training, and/or field mentorships to produce networked high-skilled African researchers. Stronger research institutions have been coupled with upcoming ones for on-going mentoring. However, results will take years to be measured, especially in Central Africa, where a critical mass of scientists is nascent and the culture of research is still dramatically lacking. Some NoEs experienced challenges in grant management, including inadequate prior establishment of monitoring and evaluation systems and delayed disbursement of funds. For instance, it was extremely difficult for EACCR to disburse funds to Sudan, possibly because of political concerns there. Some observed gaps in grant management should be filled with additional training and technical assistance in project and financial management.

### Networking

First, institutions within each NoE have learnt to collaborate in joint proposal development and project implementation, including budget sharing while avoiding duplication of effort. Second, regional NoEs have developed enriching relationships among themselves. EACCR welcomed CANTAM students and staff for short-term training on several occasions, thus reinforcing the links between institutions from the different NoEs. However, more initiatives engaging all the four NoEs should be encouraged. Third, all NoEs have interacted with various networks, organizations or northern partners, including WWARN, Global Health Trials, THRiVE, Initiative to Strengthen Research Capacity in Africa, Africa AIDS Vaccine Partnership and the African Network on Drugs and Diagnostics Innovation among others. More importantly and for purposes of sustainability, the networks are strongly encouraged to engage the policy-makers and the pharmaceutical industry to: inform policies and financing, drive health product development, best health practices and interventions [4,5,29-32].

### Advocacy

All four NoEs created their own websites to strengthen communication, interaction, advertising of training opportunities, sharing of pictures related to specific activities of the networks, and listing publications derived from research activities. Publications in peer-reviewed journals derived from baseline studies will enable each NoE to gain more visibility and credibility for sustainability.

However, frequent Internet disconnections are challenging. Communication can be improved through better Internet connectivity as recently developed by WANETAM and through more efficient video conferencing and webinar series.

### Funding

The networks have made concerted effort to leverage additional funds from alternative sources for their sustainability. TESA has performed superbly in attracting a 10-fold increase in additional funds from the baseline grant awarded by the EDCTP. Other networks are encouraged to emulate this sustainability strategy.

## Conclusion

The most rewarding experience emerging from this initiative is that the 4 networks have great potential to contribute synergistically to the attainment of the MDG 4, 6, and 8 in Africa, which focus on reduction of child mortality, combating HIV/AIDS, malaria, and other diseases for development as emphasized by a similar initiative by Wellcome Trust [24,29]. For sustainability, the NoEs recommend i) financing of streamlined career pathways, such as re-entry grant fellowships and competitive scientific awards for productive retention of young and promising African scientists, and ii) increased financial support from the African funding agencies and African governments [5,29-32] in the context of country– and regional-owned initiatives to facilitate faster progress towards achieving the MDGs on the continent.

## Competing interest

Co-authors contributed to both the implementation and the quasi-appraisal of the research networks.

## Authors’ information

The annexed team of the NoEs’ programme in Table 1, sponsored by the European and Developing Countries Clinical Trials Partnership (EDCTP).

## Pre-publication history

The pre-publication history for this paper can be accessed here:

http://www.biomedcentral.com/1471-2458/13/258/prepub
